# Safety and Clinical Benefits of Laryngeal Closure in Patients with Amyotrophic Lateral Sclerosis

**DOI:** 10.1007/s00455-022-10454-0

**Published:** 2022-05-04

**Authors:** Sayaka Yokoi, Naoki Nishio, Takashi Maruo, Mariko Hiramatsu, Nobuaki Mukoyama, Hidenori Tsuzuki, Akihisa Wada, Naoki Atsuta, Daisuke Ito, Takashi Tsuboi, Gen Sobue, Masahisa Katsuno, Yasushi Fujimoto, Michihiko Sone

**Affiliations:** 1 Department of Otorhinolaryngology, Nagoya University Graduate School of Medicine, 65, Tsurumai-cho, Showa-ku, Nagoya, Aichi 466-8550 Japan; 2 Department of Neurology, Aichi Medical University, 1-1 Yazakokarimata, Nagakute, Aichi 480-1195 Japan; 3 Department of Neurology, Nagoya University Graduate School of Medicine, 65, Tsurumai-cho, Showa-ku, Nagoya, Aichi 466-8550 Japan; 4 Aichi Medical University, 1-1 Yazakokarimata, Nagakute, Aichi 480-1195 Japan; 5 Department of Otorhinolaryngology, Head and Neck Surgery, Aichi Medical University, 1-1 Yazakokarimata, Nagakute, Aichi 480-1195 Japan

**Keywords:** Laryngeal closure, Amyotrophic lateral sclerosis, Swallowing function, Clinical benefit, Chronic aspiration

## Abstract

This study evaluated the safety of laryngeal closure and post-surgical changes in swallowing function of patients with amyotrophic lateral sclerosis (ALS) and proposed an appropriate surgical strategy for patients with ALS. Clinical and surgical data of 26 consecutive patients with ALS who underwent laryngeal closure at Nagoya University Hospital in Japan between 2003 and 2020 were retrospectively analyzed. Changes in swallowing functions were evaluated before and approximately 1 month post-surgery using Neuromuscular Disease Swallowing Status Scale (NdSSS), and Functional Oral Intake Scale (FOIS). The median operation time was 126 min (range, 51–163 min), and the median intraoperative blood loss was 20 mL (range, 0–88 mL). Among the 26 ALS patients who underwent laryngeal closure, grade 1 (mild) complications occurred in three patients (12%); however, no severe complications were observed. After surgery, 25 patients (96%) maintained the swallowing function and only one patient (4%) had deteriorating NdSSS and FOIS scores. No patients were referred to our hospital due to severe aspiration pneumonia after the surgery. Two patients did not require a feeding tube after the surgery and returned to oral intake. Laryngeal closure may be a safe surgical procedure for preventing chronic aspiration and may also maintain swallowing function of patients with ALS. Further multicenter prospective studies using the gold standard videofluoroscopic swallowing examination are required to support our findings.

**Supplementary Information:**

The online version contains supplementary material available at 10.1007/s00455-022-10454-0.

## Introduction

Amyotrophic lateral sclerosis (ALS) is a rapidly progressive and fatal neurodegenerative disease. Although various motor functions gradually worsen, few efficacious therapeutic options are currently available. Bulbar function plays a major role in determining survival outcomes, and bulbar-onset disease is associated with worse prognosis than spinal onset [1]. Malnutrition in the early stage is an independent predictor of survival in patients with ALS, and weight loss is correlated with shorter survival times [2, 3]. Furthermore, Tabor et al. assessed the effect of swallowing impairment on quality of life (QOL) of patients with ALS and highlighted the importance of early multidisciplinary interventions to improve the patients’ QOL [4]. Therefore, appropriate nutritional interventions are essential for patients with ALS to prolong survival and enhance the patients’ QOL [5].

Several surgical interventions have been reported for patients with neuromuscular diseases to improve swallowing function and enable patients to enjoy oral intake. For example, injections of botulinum toxin into the cricopharyngeal muscle [6] and cricopharyngeal myotomy [7, 8] have demonstrated positive results in patients with neurogenic dysphagia. However, these interventions have been performed mainly for patients with relatively stable disease status such as those with brain stroke or post-traumatic encephalopathy.

Patients with advanced ALS often require respiratory support with ventilators and frequent removal of secretions through tracheostomy cannulas. Chronic aspiration is virtually inevitable in patients with advanced ALS, which lowers the QOL of both patients and their families. Since Lindeman first reported the effectiveness of tracheoesophageal anastomosis for dysphagia in 1975 [9], several aspiration prevention surgeries have been proposed, including total laryngectomy [10], tracheoesophageal diversion [9], and laryngeal closure [11, 12]. Aspiration prevention surgery for severe dysphagia can improve the frequency of tracheal suction, and improvement of chronic cough and nocturnal insomnia can ameliorate patients’ depressive mood problems [13, 14]. Among these aspiration prevention surgeries, laryngeal closure is an appropriate choice of treatment for patients with irreversible severe dysphagia, such as patients with head and neck cancer, or neuromuscular disease, as laryngeal closure does not require anastomosis of the pharyngeal mucosa and can be performed even under local anesthesia. Recent studies have demonstrated that laryngeal closure in patients with severe dysphagia reduced the frequency of sputum suction and that the mood of the family members or caregivers was significantly improved after the surgery. [15] However, to the best of our knowledge, no studies on patients with ALS have investigated the safety of laryngeal closure and clinical benefits, such as prevention of aspiration and improvement in swallowing function. Thus, the management of patients with ALS who suffer from chronic aspiration remains controversial. The surgical indications depend on several clinical factors, such as ALS phenotype, preoperative swallowing function, age, general condition, and prognosis. This study aims (1) to evaluate the safety of laryngeal closure and post-surgical changes in swallowing function by reviewing our surgical experiences for patients with ALS and (2) to propose an appropriate surgical strategy for patients with ALS who plan to undergo laryngeal closure.

## Methods

### Patients

The clinical data of 26 consecutive patients with ALS who underwent laryngeal closure at our institution between 2003 and 2020 were retrospectively reviewed. The patients were diagnosed according to the revised El Escorial criteria for ALS diagnosis by experienced neurologists [16]. The ALS Functional Rating Scale-Revised (ALSFRS-R) is a commonly used and specific scale for patients with ALS and a validated rating instrument used to monitor disability progression [17]. Clinical and surgical results, including physical status, swallowing function, operative data, and complications, were analyzed.

This study was approved by the Ethics Review Committee of Nagoya University Hospital (2020–0636) and was performed according to the Helsinki Declaration of 1975, its amendments, and the Ethical Guidelines for Medical and Health Research Involving Human Subjects by the Japanese government. Requirement of consent was waived due to the retrospective nature of the study.

### Perioperative Surgical Management

**Fig. 1:**
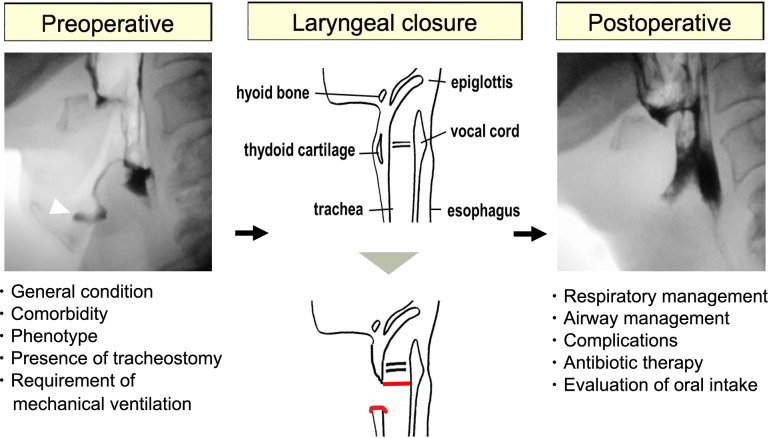
Our surgical workflow for patients planning to undergo laryngeal closure at our hospital during the perioperative period

Figure 1 shows the surgical workflow during the perioperative period for patients planning to undergo laryngeal closure at our hospital. Preoperatively, the patients were divided into two onset-based types of ALS: bulbar-onset ALS (b-ALS) and spinal-onset ALS (s-ALS) [18]. To assess a patient’s preoperative health status, we used the American Society of Anesthesiologists Physical Status (ASA-PS) [19], in which ASA-PS ≥ 3 indicates a high risk for surgery. The method of anesthesia was determined by considering the patient’s condition, comorbidity, and preference. Patients with ALS whose general condition was severe or who declined to receive prolonged mechanical ventilation in the perioperative period underwent surgery under local anesthesia.

**Fig. 2:**
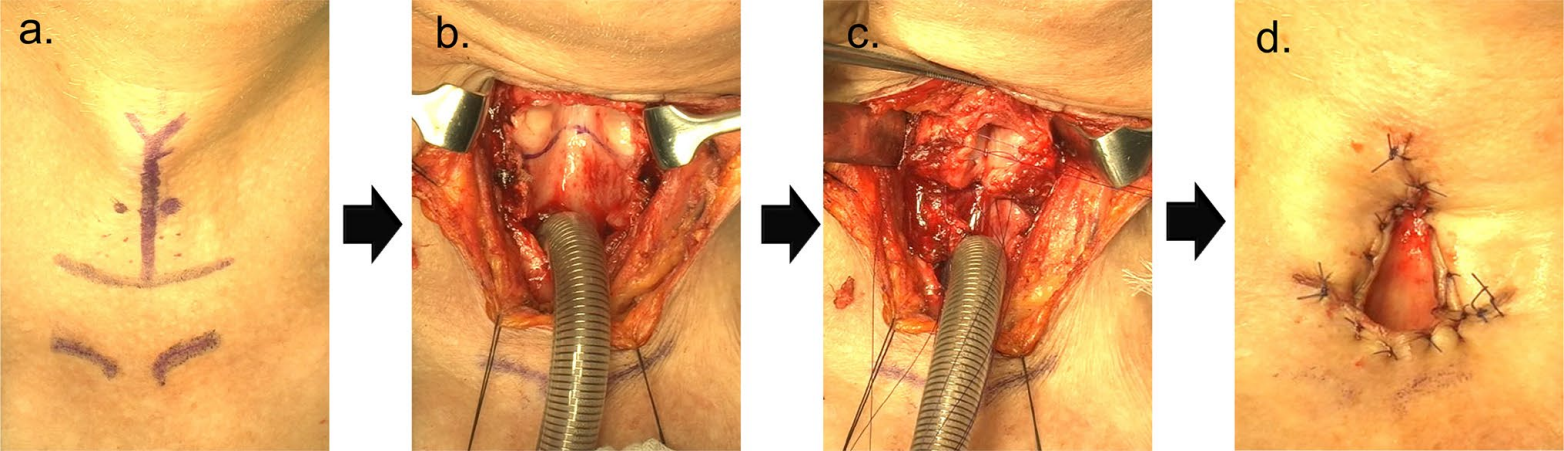
Surgical procedures in laryngeal closure **a** Incision line. **b** Removal of the thyroid cartilage, cricoid cartilage, and laryngeal opening. **c** Separation of the larynx. **d** Permanent tracheal stoma

In the operating room, patients with ALS underwent laryngeal closure under general or local anesthesia. Our surgical procedures are shown in Fig. 2. To prevent postoperative complications such as prolonged leakage of secretion from the upper edge of the larynx, we used preferentially the Kano method for laryngeal closure [20]. The anterior parts of the thyroid and cricoid cartilage were widely removed, and the glottis was closed with incised bilateral vocal folds. Finally, a sternohyoid muscle flap was used to cover the surgical defect.

After the surgery, the tracheostomy cannula was placed through the permanent tracheal hole to prevent the aspiration of the secretion and blood from the wound. The tracheostomy cannula was removed on the day after the surgery in patients who did not require continuous respiratory support with ventilators. Patients who required postoperative respiratory management with a ventilator were treated in the intensive care unit on the day of surgery. To prevent surgical site infections, antibiotics were intravenously administered for 3–7 days after surgery. Oral intake was initiated approximately 7–10 days after surgery. Voice rehabilitation is often difficult in patients with ALS who plan to undergo laryngeal closure due to the poor condition. However, an electronic artificial larynx is offered as substitute voice in selected cases.

### Complications

Postoperative complications were defined using the Clavien-Dindo classification, which is widely used to evaluate the severity of surgical complications [21]. Grade 1–2 complications are minor and require no surgical interventions; grade 3 are major complications requiring surgical intervention; grade 4 are life-threatening complications; and grade 5 indicate the death of patients.

### Swallowing Function Scale

The Neuromuscular Disease Swallowing Status Scale (NdSSS) is an evaluation scale for dysphagia in patients with progressive neuromuscular diseases and demonstrates sufficient reliability, validity, and responsiveness in patients with ALS [22]. NdSSS is rated on an 8-point scale that reflects frequently encountered clinical situations rather than the patients’ capabilities [22]. The Functional Oral Intake Scale (FOIS) is a dysphagia rating scale developed for non-progressive diseases such as stroke [23]. It is a 7-point ordinal scale used to document the functional level of oral intake of food and liquids among patients with stroke [23].

### Statistical Analyses

Descriptive statistics were performed and figures were created using GraphPad Prism (version 6.0c, GraphPad Software, La Jolla, CA, USA). Comparisons of swallowing function scale (NdSSS and FOIS) scores between the pre- and postoperative (approximately 1 month after surgery) periods were conducted using the Wilcoxon signed-rank test. All data are presented as median (range). Between patients with b-ALS and s-ALS, clinical characteristics were compared using Fisher’s exact test or the Wilcoxon signed-rank test. Statistical significance was set at a two-sided *p*-value < 0.05. (*p < 0.05; **p < 0.01; ***p < 0.001; NS not significant).

## Results

### Patients

Table 1 summarizes the clinical characteristics of the 26 patients identified (18 men, eight women). The ALS phenotype was b-ALS and s-ALS for 15 and 11 patients, respectively. The ASA-PS score was ≥ 3 for 81% of the cohort. Twenty (77%) and six (23%) patients underwent surgery under general and local anesthesia, respectively. Before surgery, one patient had cognitive impairment and two patients had severe aspiration pneumonia and required intensive treatment in the intensive care unit.

**Table 1:** Patients’ characteristics

Cases (*n* = 26)	Number
Age (years)	
Median (range)	65 (45–84)
Sex	
Male	18
Female	8
Body mass index (kg/m^2^)	
Median (range)	17.6 (12.4–26.2)
ALS types	
Bulbar (b-ALS)	15
Spinal (s-ALS)	11
Duration from disease onset to the surgery (months)	
Median (range)	31 (5–110)
Preoperative tracheostomy	
Yes	11
No	15
ASA	
1	0
2	5
3	17
4	4
Albumin (g/dL)	3.6 (2.3–4.5)
Anesthesia	
General	20
Local	6

*ALS* Amyotrophic lateral sclerosis, *ASA* American Society of Anesthesiologist

### Surgical Results

The median operation time was 126 min (range, 51–163 min), and the median intraoperative blood loss was 20 mL (range, 0–88 mL). No severe complications, including grade 3 or 4 complications, were observed. Among the 26 patients, grade 1 complications occurred in three patients (12%). One patient with bleeding from the wound bed underwent hemostasis at the bedside. Two other patients had surgical site infections owing to prolonged leakage and required antibiotic therapy (Table 2). All three patients with complications were discharged after receiving appropriate treatment. Twelve patients (46%) required mechanical ventilation after the surgery, compared to eight patients (30%) before the surgery.

**Table 2:** Complications associated with laryngeal closure

Clavien-Dindo classification	Number of patients
No complications	23
I	3
Surgical site infection	2
Bleeding	1
II-V	0

The median duration from surgery to oral food intake was 12 days (range, 4–30 days). Nineteen patients (73%) used a feeding tube with/without oral intake after the surgery, compared to 20 patients (77%) before the surgery. Notably, two patients, one with b-ALS and one with s-ALS, did not require a feeding tube after the surgery and returned to oral intake. One patient with b-ALS needed a feeding tube after the surgery because of rapid disease progression. Most importantly, no patients were referred to our hospital due to severe aspiration pneumonia after the surgery.

### Changes in NdSSS and FOIS Scores Among the ALS Phenotypes

**Fig. 3:**
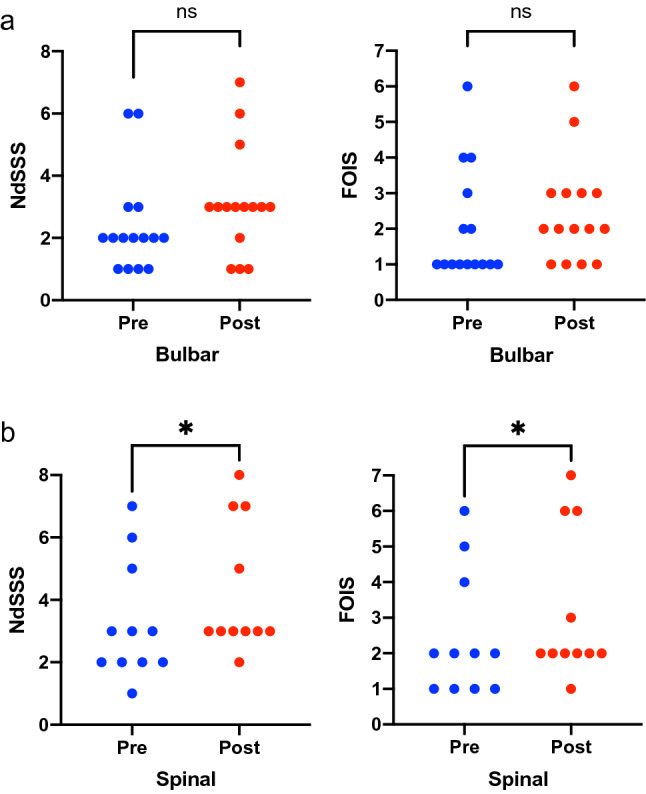
Comparison of NdSSS and FOIS scores before and after surgery in patients with the b-ALS type (**a**) and the s-ALS type (**b**). ALS, Amyotrophic lateral sclerosis; NdSSS, Neuromuscular Disease Swallowing Status Scale; FOIS, Functional Oral Intake Scale; b-ALS, Bulbar amyotrophic lateral sclerosis; s-ALS, Spinal amyotrophic lateral sclerosis

After surgery, 25 patients (96%) maintained the swallowing function and only one patient (4%) with b-ALS type had deteriorating NdSSS and FOIS scores. Patients with the b-ALS type were significantly older (*p* = 0.048) and had significantly shorter durations from disease onset to surgery (*p* = 0.0003) than those with the s-ALS type, as shown in Table 3. Figure 3 shows the changes in NdSSS and FOIS scores before and after the surgery between the b-ALS and s-ALS types. NdSSS and FOIS scores significantly improved after surgery in the s-ALS type (*p* = 0.02, *p* = 0.03, respectively). The raw data of the swallowing score among the 26 patients is shown in Supplemental Table1.

**Table 3:** Comparison of clinical characteristics in patients with the b-ALS and s-ALS types

Characteristics	b-ALS (*n* = 15)	s-ALS (*n* = 11)	*p value*
Age (years)			
Median (range)	68 (45–82)	59 (45–75)	0.048
Sex			
Male	11	7	0.68
Female	4	4	
Duration from disease onset to surgery (months)			
Median (range)	18 (5–39)	52 (10–110)	0.0003
Body mass index (kg/m^2^)			
Median (range)	17.3 (12.4–26.2)	18.3 (15.8–24.3)	0.37
Albumin (g/dL)			
Median (range)	3.9 (2.9–4.5)	3.6 (2.3–4.2)	0.075
Surgical complications			
Yes	2	2	0.99
No	13	9	

*b-ALS* Bulbar amyotrophic lateral sclerosis, *s-ALS* Spinal amyotrophic lateral sclerosis

### Changes in NdSSS and FOIS Scores Based on the Preoperative Swallowing Function

**Fig. 4:**
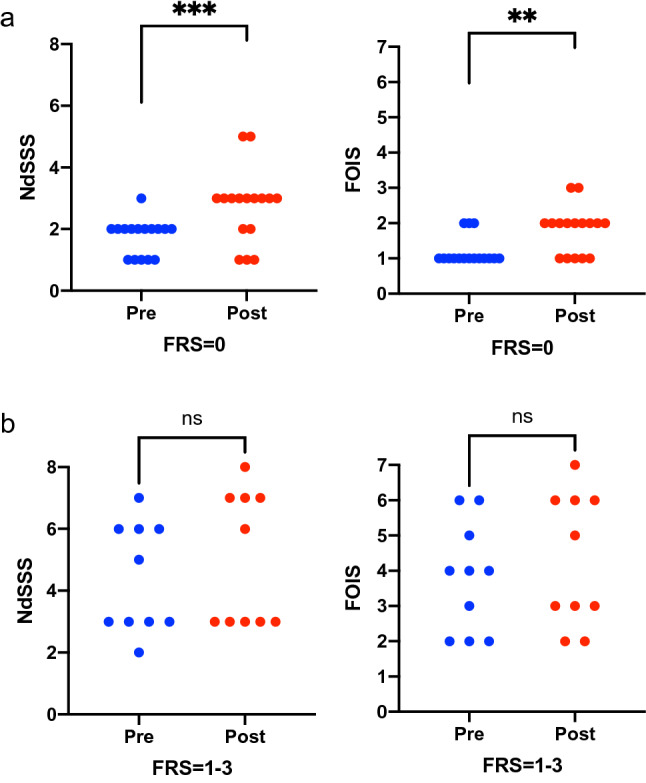
Comparison of NdSSS and FOIS scores before and after surgery in the “No oral intake” group (**a**) and “Oral intake with eating problems” group (**b**). ALS, Amyotrophic lateral sclerosis; NdSSS, Neuromuscular Disease Swallowing Status Scale; FOIS, Functional Oral Intake Scale; FRSsw, Swallowing score in Amyotrophic lateral sclerosis Functional Rating Scale-Revised

Based on the preoperative swallowing score (FRSsw) in the ALSFRS-R, we divided the patients into three groups as follows: “No oral intake” (FRSsw = 0), “Oral intake with eating problems” (FRSsw = 1–3), and “Normal eating habits” (FRSsw = 4) groups. Among 26 patients with ALS, 16 were classified as having “No oral intake” and 10 were classified as having “Oral intake with eating problems.” Fig. 4 shows the changes in NdSSS and FOIS scores before and after surgery based on the preoperative swallowing function; the NdSSS and FOIS scores significantly improved after surgery in the “No oral intake” group (*p* < 0.001, *p* = 0.004, respectively). Most patients with ALS could maintain their swallowing function after surgery in both the “No oral intake” and “Oral intake with eating problems” groups.

## Discussion

In the present study, we assessed the clinical outcomes of laryngeal closure in 26 patients with ALS and demonstrated the usefulness of laryngeal closure for maintaining swallowing function after surgery using two different swallowing scales. Mild surgical complications occurred in three patients (12%), including surgical site infection and postoperative bleeding; however, there were no severe surgical complications, thereby demonstrating the safety of laryngeal closure even for patients with advanced ALS. In our study, 25 patients (96%) with ALS maintained the swallowing function after laryngeal closure and no patients were referred to our hospital due to severe aspiration pneumonia after the surgery. Given that these patients have rapid neurodegeneration, the maintenance of swallowing function can be clinically beneficial. Two patients with ALS did not need a feeding tube after the surgery and enjoyed oral intake from the mouth. Most patients with ALS in our study needed a feeding tube after the surgery; however, laryngeal closure enabled the patients to taste the food and enjoy oral intake even at the terminal stage of the disease. To the best of our knowledge, our study is the first to demonstrate the safety and clinical benefits of laryngeal closure for patients with ALS. Enjoying oral food intake may improve the QOL of both patients with ALS and their families.

**Fig. 5:**
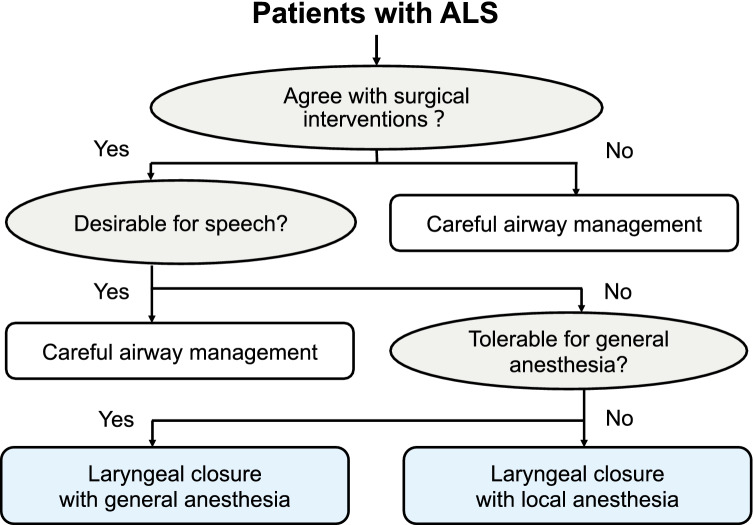
Our current surgical strategy for patients with ALS who have chronic aspiration and plan to undergo laryngeal closure. ALS, Amyotrophic lateral sclerosis

We mainly performed laryngeal closure as aspiration prevention surgery, in which the anterior parts of the thyroid and the cricoid cartilages were widely excised, and the glottis was closed by suturing bilateral vocal folds and reinforced by the sternohyoid muscle [24]. This procedure can be performed even under local anesthesia and is appropriate for patients with ALS who have poor respiratory function. We encountered six patients with ALS who did not tolerate general anesthesia or did not expect to receive mechanical ventilation during the perioperative period. To avoid unnecessary and prolonged mechanical ventilation after surgery, we performed laryngeal closure for these patients under local anesthesia without any adverse events during the perioperative period. Figure 5 shows our current surgical strategy for patients with ALS who have chronic aspiration of secretion and plan to undergo laryngeal closure. Based on patients’ preferences, prognosis, and general conditions, physicians should carefully determine the appropriate treatment option.

Bulbar function plays a major role in determining the prognosis in patients with ALS, and bulbar symptoms are indicators of poor prognosis [1, 25]. Patients with ALS can be divided into b-ALS and s-ALS types based on the body region where the first symptom appeared. Recent data from patients with ALS showed that inefficient swallowing was more frequent than unsafe swallowing (73% vs. 48%) and s-ALS type had a greater proportion of safe swallowers when compared to b-ALS type [26]. In our study, laryngeal closure could achieve a maintenance in swallowing function for most patients in both the s-ALS and b-ALS types. However, the function in one patient with the b-ALS type deteriorated after surgery. The reason for swallowing insufficiency after laryngeal closure in the b-ALS type appears to be as follows: (1) reduced pharyngeal constriction, (2) language/cognitive impairments, and (3) decreased laryngeal sensory impairment. Reduced pharyngeal constriction has been identified as a mechanism contributing to the accumulation of post-swallow residue and swallowing inefficiency in patients with ALS [27]. Several manometric studies have demonstrated a progressive decrease in pharyngeal constriction, particularly in patients with the b-ALS type [28, 29]. Conversely, Higo et al. failed to detect differences in pharyngeal pressure during swallowing between patients with s-ALS and healthy controls [30]. Moreover, language/cognitive impairments and sensory deficits of the larynx are more frequent among patients with the b-ALS type [18, 31]. We observed that some patients with the b-ALS type did not eat food after laryngeal closure because of disease progression. In the present study, patients with the b-ALS type were significantly older than those with the s-ALS type (68 vs. 59 years, *p* = 0.048), suggesting that age is an important factor for swallowing function in patients with advanced ALS.

Tracheostomy is commonly performed for patients with ALS in clinical settings for mechanical ventilation to support respiratory function and ease of suction of secretions from the lower respiratory tract. A previous study from Japan revealed that tracheostomy/mechanical ventilation was performed in 33% of patients with ALS, and it prolonged survival time compared with a non-ventilation-supported control group [32]. Moreover, a prospective longitudinal observational study using a multicenter registry revealed that there was a significant difference of approximately 7 years in life expectancy between Japanese patients with ALS who did and did not receive tracheostomy invasive ventilation therapy [33]. As mentioned above, tracheostomy is a useful and effective surgical procedure to support a patient’s respiratory condition. However, even after tracheostomy, patients had difficulty swallowing saliva, and chronic and prolonged aspiration was not completely avoidable. Laryngeal closure reduces the need for frequent suction of secretion, especially in midnight care, and prevents aspiration pneumonia, which is very important for better QOL of patients with ALS and their families, especially even among those with the advanced stages of the disease. Considering ALS phenotypes, preoperative swallowing function, age, and general condition, the surgical indication and the optimum timing for laryngeal closure should be carefully determined by patients, their families, and a multidisciplinary team.

The main limitation of this study is the relatively small number of patients with ALS from a single center based on retrospective results. Moreover, long-term follow-up results are lacking, including the prognosis or eating status after surgery. Therefore, the results need to be confirmed in larger multicenter prospective studies. ALS-FRSsw did not demonstrate adequate diagnostic accuracy to detect radiographically confirmed swallowing impairment during the screening of dysphagia in patients with ALS [34]. The Eating Assessment Tool-10 (EAT-10), a validated, self-administered, symptom-specific dysphagia outcome tool that has been implemented in clinics worldwide, could differentiated safe vs. unsafe swallowing in patients with ALS [35]. Further prospective research using the gold standard videofluoroscopic swallowing examination is required to support our findings. Furthermore, additional predictive tools, including manometric data [28] and tongue strength data [36], should be prospectively evaluated.

## Conclusion

Laryngeal closure may be a safe surgical procedure to prevent chronic aspiration and may also maintain swallowing function in patients with ALS. Further multicenter prospective studies with the use of the gold standard videofluoroscopic swallowing examination are needed to support our findings.

## Supplementary Information

Below is the link to the electronic supplementary material.

Supplementary file1 (XLSX 13 KB)

## Data Availability

All data that support the findings of this study are available from the corresponding author upon reasonable request.
